# A Novel Deployment Method for Communication-Intensive Applications in Service Clouds

**DOI:** 10.1155/2014/290913

**Published:** 2014-07-21

**Authors:** Chuanchang Liu, Jingqi Yang

**Affiliations:** State Key Laboratory of Networking and Switching Technology, Beijing University of Posts and Telecommunications, P.O. Box 187, Number 10, Xi Tu Cheng Road, Haidian District, Beijing 100876, China

## Abstract

The service platforms are migrating to clouds for reasonably solving long construction periods, low resource utilizations, and isolated constructions of service platforms. However, when the migration is conducted in service clouds, there is a little focus of deploying communication-intensive applications in previous deployment methods. To address this problem, this paper proposed the combination of the online deployment and the offline deployment for deploying communication-intensive applications in service clouds. Firstly, the system architecture was designed for implementing the communication-aware deployment method for communication-intensive applications in service clouds. Secondly, in the online-deployment algorithm and the offline-deployment algorithm, service instances were deployed in an optimal cloud node based on the communication overhead which is determined by the communication traffic between services, as well as the communication performance between cloud nodes. Finally, the experimental results demonstrated that the proposed methods deployed communication-intensive applications effectively with lower latency and lower load compared with existing algorithms.

## 1. Introduction

Web service is the major standard to realize service oriented architecture (SOA), and it encapsulates business processes into services. Based on this technology, the service platform has become a very popular way to provide services to users. However, service platforms have some disadvantages such as long construction periods, low resource utilizations, and isolated constructions. At the initial stage of service platforms, it is difficult to estimate the traffic of services to deploy physical infrastructures. Moreover, it postpones loading infrastructures to deal with sudden surge requests of service platforms. For solving these cases, the technology of cloud computing is the best choice which liberates service providers from deploying physical infrastructures. Cloud computing has achieved great commercial success in recent years. The construction and operation of extremely large-scale, commodity-computer datacenters are the key necessary enablers of cloud computing [1]. The services can be dynamically configured (via virtualization or other approaches) and delivered on demand in clouds [2]. Many cloud providers, such as Amazon EC2 [3] and Google App Engine [4], provide on-demand computing power and storage capacities dynamically. Cloud nodes of most cloud providers are large-scale located in different places.

Service clouds are distributed infrastructures which are designed to facilitate rapid prototyping and deployment of adaptive communication services in clouds [5], and they are good choices when service platforms' workloads are dynamic or they need a lot of resources. In previous work, an autoscaling mechanism to scale virtual resources in service clouds was proposed [6]. Then it is critical to find an effective method to deploy services in these resources.

Basically, there are three kinds of methods for selecting cloud nodes to deploy services in cloud, and they are Random, Ranking and Clustering-Based [7]. The method selects cloud nodes randomly. A ranking method selects the best ones from a list of ranking cloud nodes based on their QoS (quality of service), and it is usually used for computation-intensive applications. But these two methods did not take the communication of services and cloud nodes into consideration, which is very important for deploying communication-intensive applications. The basic idea of a clustering-based method is to cluster the cloud nodes which have a good communication performance together to deploy an application. The communication performance is used to measure the communication quality between cloud nodes. In this method the communication performance is measured between cloud nodes in terms of response time. The bigger the communication performance is, the faster the cloud nodes communicate with each other. Analogously, the proposed method deploys applications in nodes which have a better communication performance.

A communication-intensive application can be divided into one or more interdependent services, and services are deployed in cloud nodes. The communication frequencies and the communication volume between different services may be different. The communication frequency represents the communication times between services every interval, and the communication volume represents the data size in each communication. If services communicate frequently and have large communication volume, these services have large communication traffic which is calculated based on the commutation frequency and communication volume. A suitable deployment method should deploy services which have larger communication traffic in cloud nodes which have better communication performance, and then the overall performance will be improved.

There are several researches for provisioning and allocating services or VM (virtual machine) instances in clouds, and most of them are offline mechanisms which can find an optimal allocation. But these methods required relatively long time and were invoked periodically, and these methods did not apply to the dynamic environment in clouds. In contrast, the online mechanism can allocate services or VM instances whenever there was an allocation request, but using an online deployment for long periods would bring the cloud to a suboptimal state which was far from the optimization goal. As a result, the proposed method combines the online development and the offline development, and it improved in flexibility and effectiveness.

The main contributions of this paper are as follows.

First, the proposed model calculated the communication overhead of a service when it was deployed in service clouds, and the communication overhead was based on the communication traffic between services and the communication performance between cloud nodes. These two aspects were very important for service deployments in service clouds. In other related works, deployment methods did not take the communication traffic into consideration, and a small number of them calculate the communication performance of cloud nodes.

Second, this paper proposed a communication-aware deployment method which combined the online deployment and the offline deployment for deploying communication-intensive applications in service clouds. The combined method can overcome the shortcomings of deployment methods in related works which use one of these two deployments only.

Finally, in experimental results, the proposed method has a better performance in flexibility and effectiveness by comparing with other methods.

The remainder of this paper is organized as follows: Section 2 shows related work of cloud deployment methods; Section 3 analyzes the deployment problem of communication-intensive applications; Section 4 proposes the architecture of our system; Section 5 introduces the communication-aware deployment method; Section 6 evaluates our method; and finally Section 7 concludes this paper.

## 2. Related Work

Several researchers have dealt with issues which are concerned with resource allocation and dynamic provisioning for deploying services or VMs in cloud environments. These researches handle a variety of usage scenarios.

Huang et al. [8] propose an online service deployment strategy based on the structural property of application service flows and a dynamic resource provisioning mechanism utilizing the estimation of future system workload. In this work the proposed approaches are elaborated when the number of VMs is larger or smaller than the amount of service types to be deployed. This method improves the performance in terms of average user request response time. MOVMPA [9] is an online mechanism for VM provisioning and allocation in clouds. The mechanism is invoked as soon as a user places a request or some VM instances already allocated become available again. When invoked, the mechanism selects users who would be allocated VM instances for the period they requested for and ensures that those users will continue using those VMs for the entire period requested. The mechanism increases the number of users served but at the cost of decreased average revenue. An opportunistic service provisioning method [10] proposes an online service replication policy that leverages the variability in VM performance, as well as the on-demand billing features of the cloud. Its objective is to minimize the service provisioning costs by keeping a lower number of faster VMs, while maintaining target system utilization. Hadji and Zeghlache [11] propose an exact modified Bin-Packing problem and a minimum cost maximum flow (MCMF) to address the dynamic resource allocation problem in cloud computing environments, and it maximizes cloud provider's revenues. An energy-efficient VM placement problem is investigated in [12], and it generates multiple copies of VMs without sacrificing the QoS. This algorithm which is based on dynamic programming and local search is provided to determine the number of VM copies and then place them on the servers to minimize the total energy cost in the cloud computing system. Energy is also regarded as the measurement criteria in some other researches [13, 14].

There are some researches about deployment problems in hybrid cloud scenarios [15, 16]. Reference [15] proposes a flexible and scalable hybrid cloud architecture as well as resource provisioning policies to choose the suitable strategy based on the desired level of QoS, needed performance, and available budget. The proposed policies take into account the workload model and the failure correlations to redirect users' requests to the appropriate cloud providers.

In the above researches, in order to achieve different goals the deploy locations of services or VMs are selected according to the resource capability, energy, failure, cost, and so on. Besides these, some methods make deploy decisions based on traffic between data centers or cloud nodes.

An offline resource allocation algorithm [17] for distributed cloud systems is proposed to minimize the maximum distance or latency between the selected data centers. This algorithm selects the racks and servers where the requested VMs for the task will be located within each data center. Postcard [18] formulates an online optimization problem to minimize operational costs on inter-data center traffic, and intermediate nodes are able to store incoming data and forward them at a later time to reduce peak traffic demands. To solve the optimization problem with an acceptable number of variables, postcard has simplified the general problem by restricting data transmission to a time-slotted model, such that the problem can be modeled on a time-expanded graph. But the drawback of these methods is that they do not analyze the communication between services inside an application. Our method also takes the communication performance between cloud nodes into consideration when we deploy communication-intensive applications. Furthermore, we take the communication traffic into consideration to achieve a better performance.

Through the study of related deployment methods we can find out that there are different methods to achieve their respective goals in different scenarios. Some of the deployment methods are online, while some of them are offline. Typically user requests may arrive and leave at any time. Online methods are needed to handle the dynamic requests. However, when applications are deployed in an online manner for a long time, the performance of them may be affected. Periodically, a cloud should perform offline methods by running live migrations to bring those services or VMs together, and the cloud performance will be improved. So we combine these two methods.

In our work, we solve the issue of deploying services of a communication-intensive application in service clouds. We combine the online-deployment method and the offline-deployment method mentioned above to dynamically and effectively deploy services. For the characteristics of the communication-intensive application, each service is deployed according to communication traffic between services and communication performance between cloud nodes.

## 3. Problem Analysis

There is a large amount of communication between services in a communication-intensive application, and this is different from other types of applications. So previous deployment methods which are based on costs, energy, or computing power are no longer applicable. It is not appropriate if we do not take the communications between services and cloud nodes into consideration when we deploy the communication-intensive application in a service cloud. It will be desirable to deploy services with larger communication traffic in cloud nodes with better communication performance.

In order to deploy a communication-intensive application in service clouds appropriately, we need to know communication traffic between services and communication performance between cloud nodes.

### 3.1. Communication Traffic between Services

A communication-intensive application is composed of one or more interdependent services and communication traffic between services are usually different. We take a multimedia conference application which is communication-intensive as an example to illustrate our method in the remainder of this paper, and we also take this conference application to analyze how to obtain the communication traffic of services. Our method also can be used in other communication-intensive applications.

**Figure 1:**
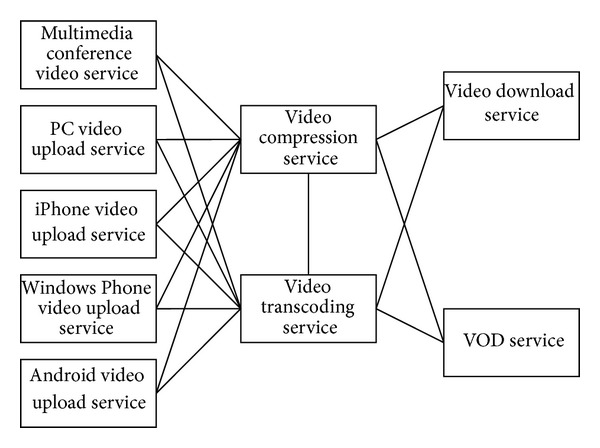
Communication relationship between services.

The multimedia conference application is developed in our laboratory, and it is a SOA-based framework. There are 9 communication-intensive services in this application, such as video upload service, video compression service, video transcoding service, and VOD (video-on-demand) service. The communication relationships of these services are shown in Figure 1. These services collaborate with each other to implement the function of the conference application. The communication traffics of each pair of services are different. For example, because the users of iPhone are more than Windows Phone, at most of the time the communication traffic between iPhone video upload service and video transcoding service is bigger than the communication traffic between Windows Phone upload service and video transcoding service. In another case, video download service does not communicate with VOD service, so the communication traffic between these two services is 0. We measure the communication traffic in terms of the average communication traffic based on monitoring data. The measuring method is shown in Section 6.

We give a formal description of the communication traffic. Assume there are* n* services in an application. The relationship of services can be represented as a* n* by* n* adjacency matrix, where each entry *x*_*i*,*j*_ represents the communication traffic from service* i* to service* j*. We do not distinguish the direction of communication in our method, so *x*_*i*,*j*_ is equal to *x*_*j*,*i*_:

(1) S1S2S3S1010S2104S3040.

As an example, we present communication traffic of an application of three services* S*1,* S*2, and* S*3 in matrix (1). Because there is no communication between* S*1 and* S*3, *x*_1,3_ and *x*_3,1_ are both 0. The communication traffic between* S2* and* S3* is more than that between* S1* and* S2* so *x*_2,3_ is bigger than *x*_1,2_.

### 3.2. Communication Performance between Cloud Nodes

In a cloud platform, we represent the cloud nodes in the form of a weighted graph. There is a path between every cloud nodes, so this graph is a complete graph. The graph is notated as *G* = (*V*, *E*), where* V* is the vertex set and* E* is the edge set. Each vertex *v*_*i*_ in* V* represents a cloud node in the cloud. Each *e*_*i*,*j*_ in* E* represents the path between two vertices. The weight of the edge *e*_*i*,*j*_ denotes the communication performance from node *v*_*i*_ to node *v*_*j*_, and it is defined by *w*_*i*,*j*_.  *w*_*i*,*j*_ is set according to the average response time during a period [19], and the concrete method is introduced in Section 6. We assume that *w*_*i*,*j*_ equals *w*_*j*,*i*_.

**Figure 2:**
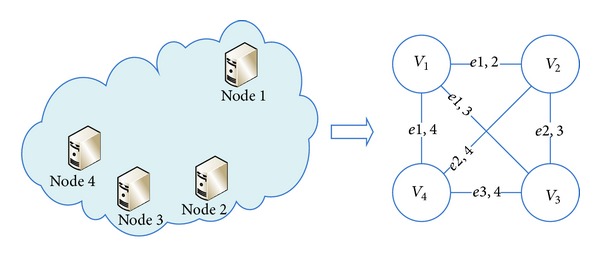
Weighted graph of cloud nodes.

In Figure 2, a sketch is shown to explain how to represent a cloud platform as a weighted graph. Suppose that there is a cloud platform which includes four cloud nodes. Every node is converted to a vertex in the right graph. Due to the underlying network topology and geographic locations of cloud nodes in the left part, the communication performance between Node 3 and Node 4 are better than that between Node 1 and Node 3. So in the right part the weight of *e*_3,4_ will be smaller than the weight of *e*_1,3_; that is, *w*_3,4_ < *w*_1,3_. If Node 2 cannot communicate with Node 3, *w*_2,3_ is set to *∞*.

## 4. System Architecture

**Figure 3:**
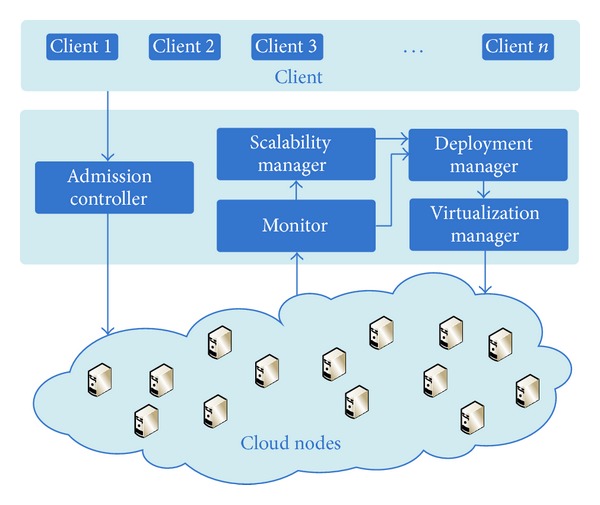
System architecture.

To implement the communication-aware deployment method for communication-intensive applications in service clouds, we design and develop a system architecture, as shown in Figure 3. Here we focus on the management of user requests and cloud nodes, so we give no more explanations on the management of network and so on. Figure 3 shows all main components which achieve the deployment of our service cloud.

*Admission Controller*. Clients generate user requests, and requests are sent to the admission controller before routing them to the services. The admission controller filtrates out invalid login requests.

*Monitor*. The monitor monitors service workloads, VM running states, communication performance of cloud nodes, and so on.

*Scalability Manager*. The scalability manager decides when and how to scale the service cloud. Based on the information collected from the monitor, the scalability manager predicts the workload at the next interval. Finally, the scalability manager proposes a scaling strategy according to platform running states and prediction results. The deployment manager executes online deployments based on these scaling strategies. The detailed method is described in [6].

*Deployment Manager*. The deployment manager is the core component for deploying services. It finds the communication traffic between services and the communication performance of cloud nodes based on the information collected from the monitor, and then it executes the online deployment and offline deployment for deploying communication-intensive applications in service clouds.

*Virtualization Manager*. The virtualization manager deals with cloud nodes directly. It executes deployment strategies proposed by the deployment manager, and it deploys services in selected cloud nodes.

## 5. Communication-Aware Deployment Method

To increase the paper readability, Table 1 lists the main measurement elements used throughout the deployment method.

### 5.1. Communication Overhead

We deploy service instances in an optimal cloud node, and the node is selected based on the communication overhead CO. CO is determined by the communication traffic CT between services and the communication performance CP between cloud nodes. When we are going to deploy a service instance* s* of an application in a cloud node, we calculate CO(*s*, *v*, *S*) for every available cloud node* v*. Then we choose a cloud node with the minimum CO(*s*, *v*, *S*) and this node is the optimal one. The calculation model of CO(*s*, *v*, *S*) is shown in

(2) $$\begin{array}{c} \text{CO}\left(s,v,S\right)=\underset{s_{i}\in S}{\sum }\underset{v_{i}\in v_{s_{i}}}{\sum }\text{CT}\left(s,s_{i}\right)*\text{CP}\left(v,v_{i}\right). \end{array}$$

The communication overhead CO(*s*, *v*, *S*) is the total sum of products of CT and CP. More specifically, an application is composed of a set of services *S* = {*s*_1_, *s*_2_,…, *s*_*n*_}, and every service can be deployed in one or more cloud nodes. For every service* s*_*i*_ in* S*, first we calculate CT between* s* and* s*_*i*_. Then we find out all cloud nodes *v*_*s*__*i*_ which deploy* s*_*i*_, and we calculate every CP between* v* and every node* v*_*i*_ in *v*_*s*__*i*_. Finally we add all products of CT and every CP together.

The computational complexity for calculating CO(*s*, *v*, *S*) is *O*(*m*∗*n*), where* m* is the number of services deployed in service clouds and* n* is the number of cloud nodes. In the worst case, the value may be calculated for *m*∗*n* times. When there are a lot of service instances deployed and services are deployed in many cloud nodes, this calculation may take a long time. In this case, we can divide the cloud nodes into different regions, and the algorithm is executed in each region separately, and then the value of* m* and* n* can decrease.

### 5.2. Online Deployment

When a new request to deploy a service instance is received, the online-deployment algorithm is executed to decide its allocation. Pseudocode of the online-deployment algorithm is given in Algorithm 1.

In Algorithm 1, when a deployment request of service instance* s* is received, first we obtain the set of services* S* which are in the same application with* s*. To prevent the same service instance from being deployed in the same node for the availability, service instances of the same service should be deployed in different cloud nodes. So we obtain the set of candidate cloud nodes* V* which have available space, and *s* do not deploy in these nodes. If there are available nodes (line 2), we calculate CO(*s*, *v*, *S*) for every* v *(lines 4-5). Then we find out the minimum CO (min CO) and the corresponding cloud node *v*_min⁡_ (lines 6–8). Finally we can deploy the service* s* in this cloud node *v*_min⁡_ (line 11).

### 5.3. Offline Deployment

When a new request to deploy a service instance is received, we use the online method to choose a cloud node to deploy this service, and this cloud node is the optimal one at that time. Because the overall deployment is changing all the time with new service deployments and service deletions, gradually the original deployment strategy is not an optimal one in the new circumstance. Then the system may come to a suboptimal state. As a result of this, we execute the offline-deployment algorithm every once in a while to reoptimize the deployment of services.

The more the times of service deployments service deletions are, the more frequently the offline method should be executed. This frequency can be set based on the times of new service deployments and service deletions. If the offline method is executed too frequently, it may bring a large migration overhead and the system performance may be affected. But if the offline method is executed too rarely, the system is in a suboptimal state and the performance also may be affected. We can choose the execution frequency of the offline method through an experiment. We conduct the experiments when the offline method is executed after 40, 60, 80, and 100 times of service deployments and deletions, respectively, and we record the latency and load in these cases.

**Figure 4:**
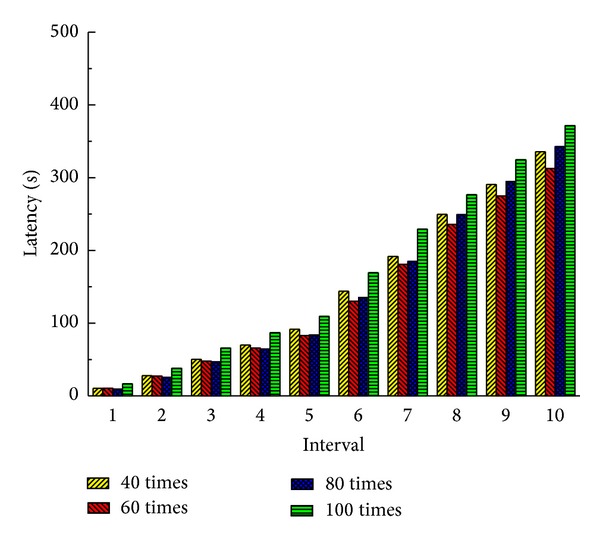
Total latency of all services (20 nodes).

**Figure 5:**
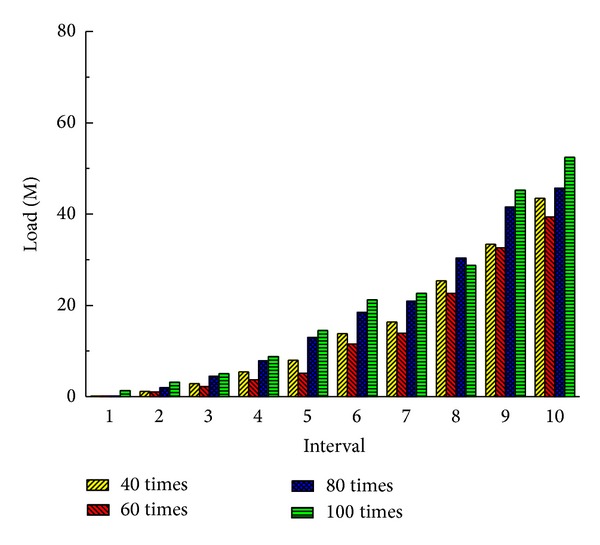
Total load of communication (20 nodes).

From Figures 4 and 5 we can see that the performance of the system is the best when the number of service deployments and deletions are 60 in our environment. So we execute the offline method every time the times of service deployments and deletions are 60.

The offline-deployment algorithm migrates service instances which can reduce the most communication overhead between cloud nodes, and it can reduce the total communication overhead to an extreme. Pseudocode of the offline-deployment algorithm is given in Algorithm 2.

The offline-deployment algorithm is executed LOOP times each time. When we set the value of LOOP, we should make a tradeoff among the computation overhead, the number of migrated service instances, and the application performance. The value of LOOP is dynamic in our experiments. Because when the max DIF is too small the service migration makes little sense, the offline method is executed until the percentage of max DIF in the total communication overhead is below a lower limit. In order to prevent the algorithm from executing too many times, we can set a maximum value of the LOOP. Then we start the iteration.

There may be one or more services running in a cloud node. So for every cloud node* v*, we calculate current CO (CurrentCO) of every service* s* running in* v* (line 4–6). Then suppose that this service can be deployed in other available cloud nodes; we calculate min CO and *v*_min⁡_ of* s* (line 7). After all the node traversals and service traversals, we can obtain the maximum difference (max DIF) between CurrentCO and min CO. Besides these we can get the corresponding service *s*_migrate_ which has max DIF, the cloud node *v*_current_ where *s*_migrate_ is deployed now, and the cloud node *v*_destination_ where *s*_migrate_ will be migrated to (lines 8–13). If there is a service whose performance can be improved, we migrate *s*_migrate_ from *v*_current_ to  *v*_destination_ (lines 16-17). Finally we decrement LOOP by one and begin the next iteration (line 19).

## 6. Evaluation

### 6.1. Experimental Preparation

We carry out the experiment simulation in a Java development environment. Experiments are designed to measure the latency and the load of our method, two transformation methods, and other four comparison methods.

*Latency*. In our experiment, the latency of a service is defined as the duration between sending out a request and receiving a response. 

*Load*. In our experiment, the load of a communication is defined as the product of the data transfer volume and the data transfer time.

First we measure communication traffic between services and communication performance between cloud nodes beforehand for the forthcoming experiment.

We monitor communication frequencies and communication volume of each communication for every two services for a period of time, and we take the product of average frequencies and average volume as the communication traffic between two services. Each communication traffic is normalized by the largest communication traffic, and the communication traffic of the same service is set to 0.

Our experimental environment provides two scenarios, and they consist of 20 and 10 distributed nodes which serve as cloud nodes. We measure the response time between every two cloud nodes periodically and use the average response time during a period as the communication performance between two cloud nodes. Each communication performance is normalized by the largest communication performance, and the communication performance of the same node is set to 0.

First, in order to find out the superiority of our online and offline combined method, we evaluate the performance of two transformation methods of our communication-aware method which is labeled as “CA,” and these two transformation methods are as follows.

Online method only executes the online method of our method.

Offline method only executes the offline method of our method and randomly selects cloud nodes to deploy services when there are real-time service deployment requests.

**Figure 6:**
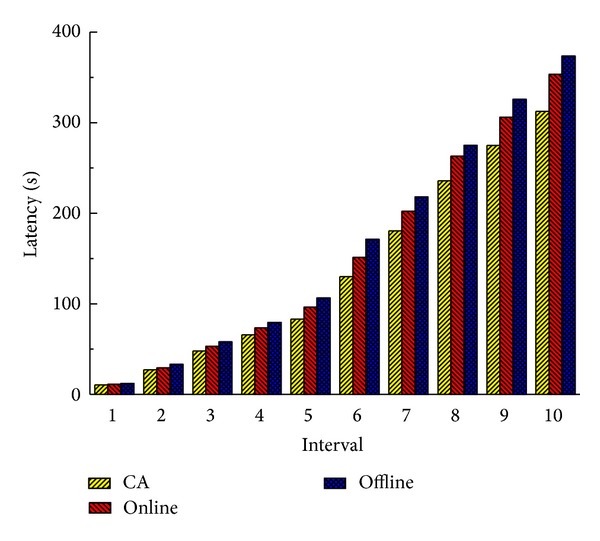
Total latency of all services (20 nodes).

**Figure 7:**
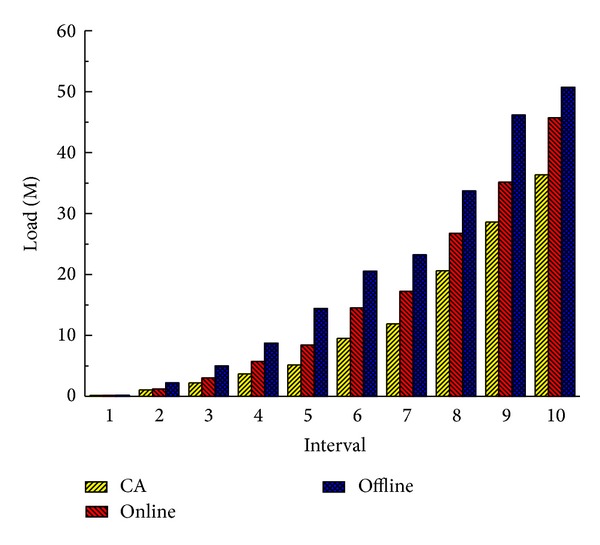
Total load of communication (20 nodes).

From the experimental results in Figures 6 and 7 we can find out that our combined method performs better than the other two transformation methods. Online method does not optimize the system when the system is in a suboptimal state. Offline method executes too many times service migrations to optimize the system, and this brings a lot of system overhead. So these two methods are not as good as CA. This illustrates that the combination of the online method and the offline method can improve the system performance.

Then, we use four comparison methods, Random, Rank, NA, and CPOnly, to compare with our method. Random and Rank methods are online-deployment methods. NA is an offline method in [17]; due to the defect of offline methods we use Random method as an online method in NA to handle real-time deployment requests. CPOnly method is a combined method of online and offline deployments. When there is a deployment request, methods will be executed repeatedly until all the services in the request are deployed.

Random method selects a random cloud node which is available and deploys a service instance in it.

Rank method selects the cloud node with the maximum computing power and deploys a service instance in it.

NA method uses an efficient 2-approximation algorithm for the optimal selection of data centers, the racks, and servers. Its objective is to minimize the maximum latency between the selected nodes.

CPOnly method is similar to our method. But it only takes the communication performance between two cloud nodes into consideration and does not consider the communication traffic between services. The communication overhead of CPOnly is calculated as

(3) $$\begin{array}{c} \text{CO}\left(s,v,S\right)=\underset{s_{i}\in S}{\sum }\underset{v_{i}\in v_{s_{i}}}{\sum }\text{CP}\left(v,v_{i}\right). \end{array}$$

### 6.2. Experimental Results

In the experiment, we execute the online deployment and the offline deployment to handle service deployment requests of the multimedia conference application. During this process, we invoke services periodically, and invocation patterns of services depend on overall communication patterns of services in the application monitored before. We calculate the latency and load between services for every interval as experimental results. For the confidence results, we collect the experimental results from multiple runs and obtain the average values as the final results.

**Figure 8:**
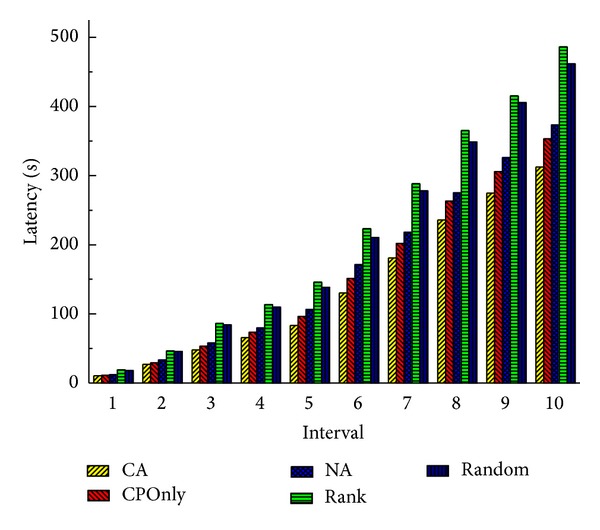
Total latency of all services (20 nodes).

**Figure 9:**
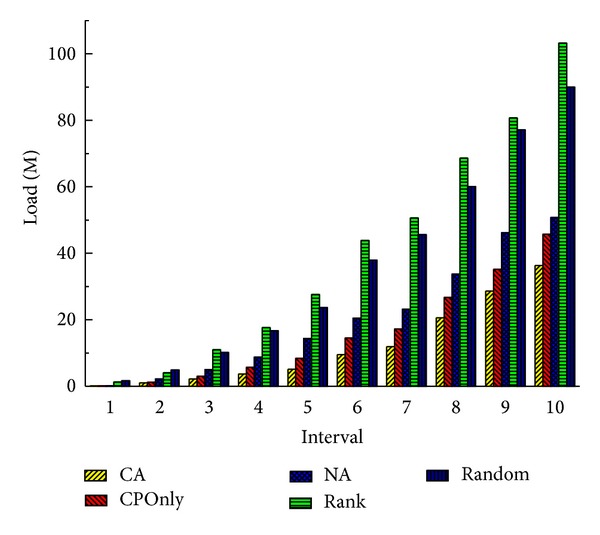
Total load of communication (20 nodes).

Figures 8 and 9 show the total latency of all services and total load of communication at 10 time intervals when there are 20 cloud nodes in the service cloud. Obviously, because Rank and Random deploy services regardless of communication, they have higher latencies and loads than CA by 47%–55% and 120%–180%, respectively, at the last interval. It is worth noting that sometimes Random performs a little better than Rank. Because Rank selects the node which has the maximum computing power, services may be dispersed in cloud more widely than Random. And this may affect the communication performance. Benefiting from the consideration of communication traffic between services, CA outperforms CPOnly by 12% and 23% in latency and load. NA also takes the communication performance into consideration, but it is an offline method; when there are real-time service deployment requests, it chooses a random cloud node to deploy services. So the system performance is affected, and it performs worse than CA in terms of latency and load.

**Figure 10:**
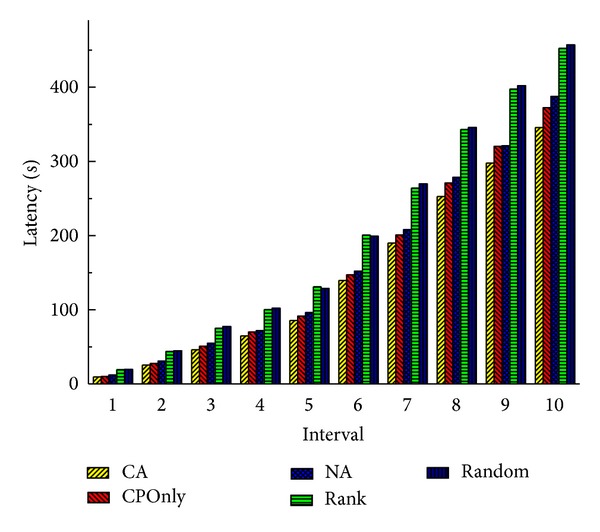
Total latency of all services (10 nodes).

**Figure 11:**
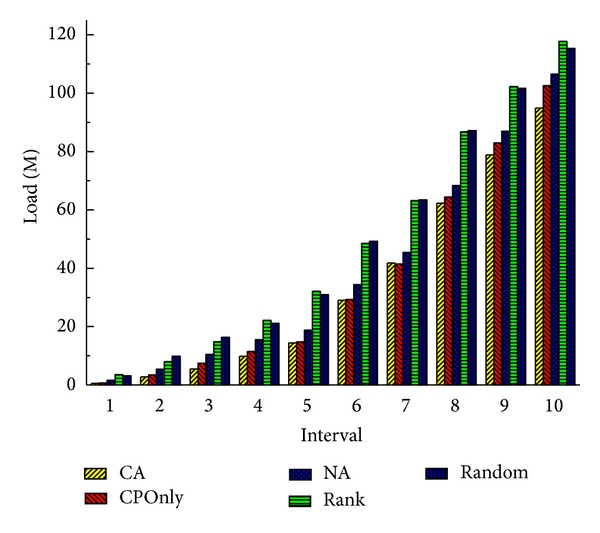
Total load of communication (10 nodes).

When the number of cloud nodes decreases to 10, we get the similar results which are shown in Figures 10 and 11. It is slightly different that differences between four methods decrease, and CA performs better than CPOnly, NA, Rank, and Random in latency and load by 7%, 12%, 31%, and 32% and 8%, 13%, 24%, and 22%, respectively, at the last interval. Because, with the decrease of cloud nodes, the number of alternative deployment locations is also reduced, and each method is more likely to select similar nodes or even the same nodes to deploy a service. But even so our CA outperforms other methods and achieves the shortest latency and the smallest load.

In this paper, for lack of space, we do not mention how to select a cloud node and remove a service instance from it in a service cloud. The remove method also depends on the communication overhead. When we need to remove a service instance, if the service is only deployed in one cloud node, we remove it from the node directly. If there are multiple copies of the service in different nodes, we remove the service instance from one of them which has the largest communication overhead. And experimental results show that this method works well.

## 7. Conclusion

This paper investigated the problem of deploying services in service clouds. Unlike previous works, the proposed work takes communication traffic between services and communication performance between cloud nodes into consideration. In the deployment algorithm, it combined the online-deployment method and the offline-deployment method. This algorithm also combined the benefits of these two methods. It can dynamically handle deployment requests and attain a good performance. In addition, the proposed approach is compared with two transformation methods and other four comparison methods in experiments. In experimental results, the proposed method can deploy communication-intensive applications properly, and the application can gain lower latency and lower load.

## Figures and Tables

**Algorithm 1:**
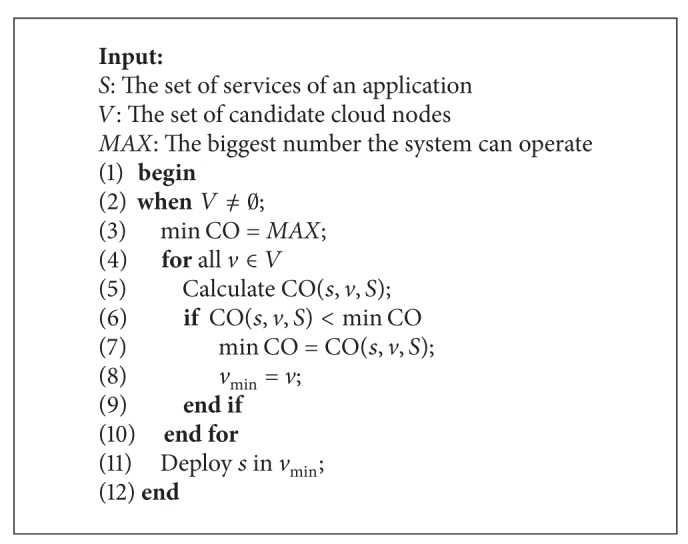
Online deployment.

**Algorithm 2:**
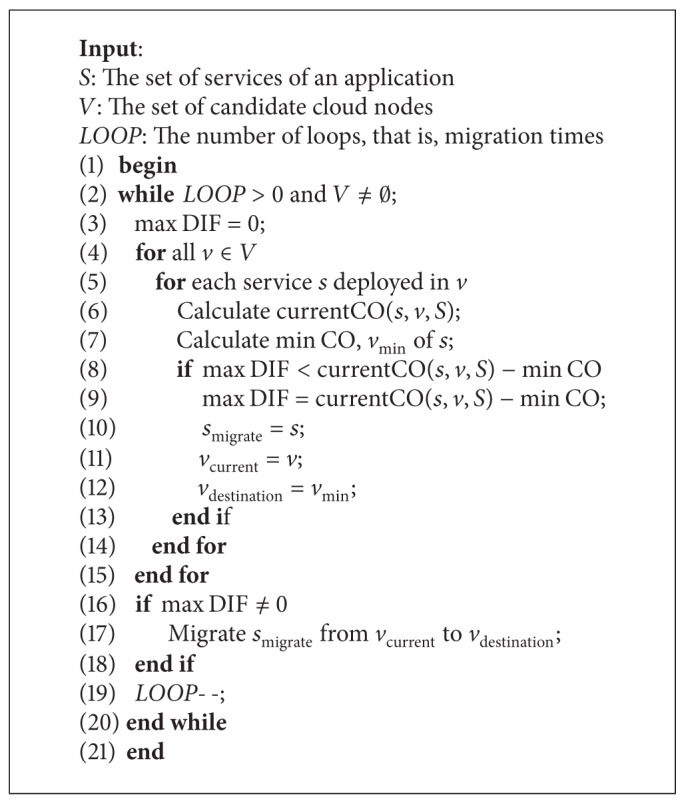
Offline deployment.

**Table 1:** Elements of the deployment method.

Element	Description
CO(*i*, *j*, *S*)	Communication overhead between service *i* and service set *S* when *i* is deployed in cloud node *j*
CT(*i*, *j*)	Communication traffic between service *i* and service *j*
CP(*i*, *j*)	Communication performance between node *i* and node *j*
